# Ramadan fasting induces temporal gut microbiome remodeling without metabolic improvement in adults with prediabetes

**DOI:** 10.1128/spectrum.00742-26

**Published:** 2026-08-03

**Authors:** Ihsan Boyaci, Busranur Delice, Fatma Koc, Suleyman Yildirim

**Affiliations:** 1 Department of Internal Medicine, Istanbul Medipol University Faculty of Medicine, Istanbul, Türkiye; 2 Department of Medical Microbiology, Istanbul Medipol University International School of Medicine, Istanbul, Türkiye; 3 Regenerative and Restorative Medicine Research Center, REMER, Istanbul Medipol University & Research Institute for Health Sciences and Technologies (SABITA), Istanbul Medipol University, Istanbul, Türkiye; NC State University, Raleigh, North Carolina, USA

**Keywords:** prediabetes, Ramadan fasting, gut microbiome dynamics, metabolic parameters, time-restricted feeding

## Abstract

Ramadan fasting is a naturally occurring, time-restricted dietary pattern involving daily abstinence from food and drink from dawn to sunset. In this longitudinal study, we investigated the impact of Ramadan fasting on gut microbiome dynamics and metabolic outcomes in prediabetic individuals, whose responses to fasting can be uniquely altered due to decreased metabolic plasticity. Seventeen adults diagnosed with prediabetes were sampled pre-Ramadan (baseline), end of Ramadan (1-month), and 3 months post-fasting. Gut microbiome was profiled using 16S rRNA gene sequencing, and metabolic and anthropometric measurements were performed. Time-dependent coordinated changes were investigated using network-based approaches. Ramadan fasting was associated with distinct, time-dependent shifts in the gut microbiome. Genus-level richness did not change significantly across time points, while alpha diversity (Shannon and Simpson indices) increased significantly at the end of Ramadan and partially reverted by 3 months post-fasting. Beta diversity analyses showed the greatest compositional separation between baseline and 1-month, followed by partial reversion at 3 months. End-of-Ramadan samples showed the enrichment of facultative anaerobes and selected short-chain fatty acid (SCFA)-associated taxa, whereas several butyrate-producing genera increased by 3 months post-fasting, consistent with temporally phased dynamics among SCFA-associated lineages. Despite these microbial and predicted functional changes, glycemic and lipid markers remained largely unchanged, while insulin resistance and adiposity indices increased over time, and microbiome-metabolic relationships did not translate into consistent metabolic improvement.

**IMPORTANCE:**

This study shows that Ramadan fasting is associated with temporal remodeling of gut microbial composition and predicted function in individuals with prediabetes although these changes were not accompanied by clear short-term metabolic improvement. We observed coordinated shifts in bacteria linked to short-chain fatty acid production and changes in overall community balance. However, these microbial changes did not translate into measurable improvements in blood glucose or lipid levels. These findings suggest that microbiome-associated changes occurring during Ramadan fasting may not be sufficient, within the context of prediabetes, to yield measurable short-term metabolic improvement. Behavioral and metabolic restrictions may influence the extent to which microbiome-associated changes correspond with metabolic outcomes, highlighting the need for larger, diet-controlled longitudinal studies.

## INTRODUCTION

Prediabetes is a significant intermediate metabolic state between normoglycemia and type 2 diabetes mellitus (T2DM), affecting more than 10% of the adult population worldwide (1). People diagnosed with prediabetes have a much higher risk of developing T2DM, heart disease, and other metabolic disorders (2).

Biochemically, prediabetes is diagnosed exclusively based on glycemic criteria, including elevated fasting plasma glucose, impaired glucose tolerance, and increased glycated hemoglobin (HbA1c) levels (3). From a pathophysiological perspective, these glycemic abnormalities are frequently accompanied by hyperinsulinemia and reduced insulin sensitivity, which can be quantified using indices such as the homeostatic model assessment of insulin resistance (HOMA-IR), although these measures are not used for diagnostic purposes.

Furthermore, disruptions in lipid metabolism, including reduced high-density lipoprotein (HDL) cholesterol levels, elevated triglycerides, and variable elevations in low-density lipoprotein (LDL), are commonly observed (4).

The gut microbiome regulates host energy homeostasis and metabolic health (5). Dysbiosis in prediabetes and T2DM has been consistently linked to lower abundance of SCFA-producing taxa, an increase in opportunistic pathogens, and functional impairments in metabolic pathways (6, 7). These changes shape host physiology by affecting intestinal barrier function and inflammatory processes. Furthermore, it has been shown that the gut microbiota can predict individual responses to dietary and lifestyle interventions (8). Targeting microbiome-host interactions in prediabetic states has been proposed as a potential strategy to modulate metabolic dysfunction and possibly delay diabetes progression.

Under non-drug interventions, intermittent fasting has received intense interest as a potential aid to metabolic well-being (9). Ramadan fasting, practiced by over one billion people annually, represents a natural pattern of daily intermittent fasting involving abstaining from food and drink from sunrise to sunset and limiting food intake to evening (iftar) and suhoor meals for a month. This pattern significantly alters meal timing, circadian rhythms, energy intake, and physical activity (10, 11). Although studies have associated Ramadan fasting with improvements in body weight, lipid profiles, blood pressure, and glycemic measurements, findings are variable and thought to depend on baseline metabolic status and dietary behaviors (12–15). Most microbiome studies related to Ramadan have focused on healthy individuals and short-term effects, with the limited assessment of microbial function or post-Ramadan improvement, particularly in individuals with prediabetes.

To address these gaps, we performed a longitudinal analysis of adults with prediabetes across three time points: pre-Ramadan, end of Ramadan, and 3 months post-fasting, integrating gut microbiome profiling, predicted functional capacity, and metabolic measurements. We hypothesized that Ramadan fasting would be associated with temporal remodeling of the gut microbiome in prediabetic adults and that these shifts may partially align with changes in host metabolic parameters. Our objective was to define temporal patterns of microbial and functional adaptation and evaluate how these patterns corresponded with metabolic indices in this metabolically vulnerable patient population.

## MATERIALS AND METHODS

### IssuesMethodology of research, and participant selection

This study was conducted in the Internal Medicine outpatient clinics of Istanbul Medipol University and Research Center after obtaining institutional ethics committee approval (approval no: 10840098-604.01.01-E.9264). Seventeen prediabetic adults aged 40–65 years, 7 male and 10 female, participated after signing written informed consent.

### Inclusion criteria for participant selection

Prediabetes was defined according to the American Diabetes Association (ADA) criteria (3), including fasting plasma glucose (FPG) 100–125 mg/dL (5.6–6.9 mmol/L), 2 h plasma glucose (2 h PG) 140–199 mg/dL (7.8–11.0 mmol/L) during a 75 g oral glucose tolerance test (OGTT), or glycated hemoglobin (HbA1c) 5.7%–6.4%. Individuals meeting any of these thresholds but not fulfilling the diagnostic criteria for diabetes were classified as having prediabetes. All participants were required to be willing and able to comply with study procedures, including biological sample collection and follow-up assessments.

### Exclusion criteria for participant selection

Exclusion criteria included chronic diseases other than type 2 diabetes, treatment with antimicrobial or immunosuppressive agents in the previous 3 months, active or recent infection, recent intestinal surgery, pregnancy or lactation, lactose intolerance, gastrointestinal disease (e.g., IBD, Crohn’s, celiac disease), substance/alcohol dependency, and any condition deemed unsuitable by the clinician. Participants were also excluded if they were unable to comply with sample collection requirements or used probiotics, prebiotics, or synbiotics within the past month. Participants were also excluded if they were unable to comply with sample collection requirements or if participation was deemed unsuitable by the attending clinician.

### Anthropometric assessment

Comprehensive medical history and physical examination were conducted in the outpatient clinic. Anthropometric measurements were obtained by trained personnel following standardized procedures. Body mass index (BMI) was calculated as weight (kg) divided by height squared (m²). Waist and hip circumferences were measured using a non-stretchable tape, with waist circumference assessed midway between the lower rib margin and the iliac crest, and hip circumference measured at the widest point over the buttocks. The waist-to-hip ratio (WHR) was calculated accordingly. According to the World Health Organization (WHO) criteria, WHR values <0.90 for males and <0.85 for females were considered within the normal range (16).

### Collection of samples and biochemical analyses

Blood and stool samples were collected from participants at three predefined time points: within 3 days before Ramadan (baseline; day 1), during the last 3 days of Ramadan (1-month), and 3 months after the end of Ramadan (3-month time point). Participants were instructed to avoid major dietary changes throughout the study period and to collect stool samples using sterile collection kits provided by the research team.

Standardized clinical and biochemical assessments were performed to evaluate glycemic status, lipid profile, renal function, and insulin-related parameters, including fasting plasma glucose, HbA1c, oral glucose tolerance test, total cholesterol, triglycerides, HDL-C, LDL-C, fasting insulin, C-peptide, and HOMA-IR. Insulin resistance was calculated using the homeostatic model assessment of insulin resistance (HOMA-IR) index: HOMA-IR = (fasting insulin (μIU/mL) × fasting glucose (mmol/L))/22.5 (17). All samples were processed and stored according to standardized protocols. Detailed descriptions of sample handling procedures, analytical platforms, assay methodologies, and calculations are provided in the Supplemental materials.

### Recovery of stool DNA and sequencing 16S rRNA genes

Stool samples were collected and stored at −80°C until processing. Microbial genomic DNA was extracted using standardized protocols, followed by amplification of the V3-V4 hypervariable regions of the 16S rRNA gene. Amplicon libraries were prepared with appropriate negative controls and sequenced on the Illumina MiSeq platform. Raw sequencing data were uploaded to the NCBI Sequence Read Archive (accession number: PRJNA1329139). Detailed descriptions of DNA extraction procedures, PCR conditions, library preparation, sequencing parameters, and quality control steps are provided in the Supplemental methods section.

### Data analyses

#### Sequencing processing and taxonomic classification pipeline

The paired-end reads were processed using DADA2 pipeline running on Nephele platform (18) (v.2.30, http://nephele.niaid.nih.gov). The steps of primer trimming, quality filtering (maxEE = 5; truncQ = 4), denoising (using default DADA2 error models), merging of paired-end reads (maxMismatch = 0), and chimera removal (enabled by default) were performed, yielding high-quality amplicon sequence variants (ASVs). To account for variations in sequencing depth, the ASV table was rarefied to a minimum library size of 15,077 reads prior to alpha diversity estimation. For all downstream analyses, ASVs were filtered using a prevalence threshold of >5% across samples and a minimum abundance threshold of 10 counts to ensure the exclusion of low-frequency and potentially spurious features. Furthermore, negative controls (*n* = 3) were processed in each sequencing run to monitor for potential contamination. While two negative controls yielded zero reads, one control (19,095 reads) was subjected to prevalence-based decontamination assessment. No contaminant ASVs exceeding the detection threshold were identified, confirming the absence of meaningful contamination within the data set. ASVs annotated as chloroplasts, mitochondria, archaea, eukaryotes, or unclassified non-bacterial features were removed prior to functional inference, and downstream analyses to ensure microbial relevance. The taxonomic classification was carried out using SILVA small subunit rRNA database (19) (v.138). For functional prediction purpose, PICRUSt2 (20) was executed locally using the SILVA-based ASV table as input to infer metagenomic functional potential. For all other analyses sensitive to data compositionality, center log-ratio (CLR) transformation was applied. Further ecological and statistical analysis, such as alpha and beta diversity estimation, testing for differential abundance, and correlation analysis, was conducted with the phyloseq R package (v.1.36.0) in R (v.4.2.3).

#### Microbial community structure and diversity analyses

Alpha diversity was assessed at the genus level using Observed richness, Chao1, Shannon, and Simpson indices. To account for the longitudinal study design with repeated measurements from the same individuals, differences in alpha diversity across fasting intervals (1-day, 1-month, and 3-month) were evaluated using Friedman repeated measures tests.

Beta diversity was calculated based on Bray-Curtis dissimilarities and visualized using ordination approaches. Overall and pairwise differences in microbial community composition across fasting intervals were tested using permutation-based multivariate analyses with subject level constraints to account for within individual dependencies.

Indicator taxa associated with specific fasting intervals and associations between microbial taxa and host metabolic parameters were examined using correlation and classification-based methods, and core microbiome analyses were performed to identify taxa consistently present across time points. All analyses were conducted in R (v4.2.3) using established microbiome analysis packages, including phyloseq, vegan, indicspecies, microbiome, WGCNA, and ggplot2, along with custom scripts. Detailed descriptions of statistical tests, model specifications, thresholds, and software implementations are provided in the Supplementaly methods.

#### Multivariable and network-based analyses

To identify microbial features associated with clinical and temporal variables, we employed MaAsLin2 (21) (Microbiome Multivariable Association with Linear Models, v1.10.0) in R. Genus-level relative abundance data were analyzed with appropriate feature filtering and transformation, incorporating both continuous (e.g., age, BMI, glycemic and lipid parameters) and categorical (fasting interval) metadata. Statistical significance was determined using false discovery rate correction.

Cross-omics relationships between microbial taxa and predicted functional pathways were explored using the MintTea framework (22) to identify robust co-occurrence modules across fasting intervals. Network-based integration was performed using iterative subsampling and multivariate correlation approaches, and modules demonstrating discriminative capacity were retained for downstream interpretation.

Consensus networks were visualized to characterize interval-specific connectivity patterns between microbial and functional features. Detailed descriptions of preprocessing steps, model parameters, validation criteria, and network construction procedures are provided in the Supplementary methods.

## RESULTS

We examined the temporal effects of Ramadan fasting on gut microbial composition and metabolic parameters in adults with prediabetes using longitudinal sampling at three time points, pre-Ramadan (day 1), end of Ramadan (1-month), and 3 months post-fasting. Genus-level 16S rRNA gene sequencing data were integrated with metabolic and anthropometric measurements. A multi-tiered analytical framework, including diversity analyses, taxonomic profiling, differential abundance testing, multivariable associations, and predicted functional inference, was applied to characterize fasting-associated changes in microbial ecology and their relationship to host metabolic indices.

### Participant characteristics

Seventeen adults with prediabetes were enrolled in the study. Baseline demographic characteristics are summarized in Table 1. The mean age of participants was 50.24 ± 5.57 years. Stool sample availability varied across sampling intervals. At baseline (day 1), a total of 19 stool samples were analyzed, reflecting technical replicate sampling (triplicate collection) from one participant.

**TABLE 1:** Baseline demographic characteristics of the study participants

Variable	Value
Participants (*n*)	17
Sex (male/female)	10/7
Age (years)	50.24 ± 5.57
Height (cm)	164.00 ± 8.34

Seventeen healthy adults (HC), matched for age and sex, were included as a reference cohort (23) for baseline cross-sectional comparison with prediabetic participants. Baseline demographic characteristics of HC participants are provided in Table S1.12. Beta-diversity analysis based on Bray-Curtis dissimilarity demonstrated significant compositional separation between HC and prediabetic baseline samples (PERMANOVA: *R*² = 0.134, *F* = 4.729, *P* = 0.008) independent of age and sex covariates (Fig. S14). Across alpha-diversity metrics, HC samples exhibited significantly higher microbial diversity compared with all prediabetic time points (BH-adjusted *P* < 0.001; Fig. S13; Table S1.10), whereas no significant alpha-diversity differences were observed among prediabetic time points. Differential abundance analysis (LEfSe) identified 62 genera differing significantly between HC and prediabetic baseline samples. Of these, 60 genera were enriched in prediabetic participants, including *Dialister*, *Escherichia-Shigella*, and *UCG-002*, whereas two genera, *Enterocloster and Subdoligranulum*, were enriched in HC samples (Fig. S17; Table A3.2). Together, these findings indicate that prediabetic individuals exhibited a distinct baseline gut microbial composition relative to healthy adults prior to Ramadan fasting.

Technical replicates (*n* = 3 from a single biological sample at the 1-month time point) exhibited high agreement, with a mean pairwise Bray-Curtis distance of 0.057 (R1–R2 = 0.074, R1–R3 = 0.073, R2–R3 = 0.025; Table S1.6; Fig. S16). This value was 10.7-fold lower than the mean biological Bray-Curtis distance across non-replicate samples (mean = 0.61), indicating that pipeline-associated technical variation was substantially smaller than the observed biological variation. Because all prediabetic samples were processed within a single sequencing batch using identical laboratory protocols, inter-run batch variability within the cohort was minimized.

Anthropometric measures showed increases in waist circumference, waist-to-hip ratio, and fat mass at both 1 and 3 months relative to baseline (Table 2).

**TABLE 2:** Longitudinal anthropometric measurements before, during, and after Ramadan fasting^*b*^

Variable	Before Ramadan (1-day)	End of Ramadan (1-month)	After Ramadan (3 months)	*P* value^*a*^
Weight (kg)	82.98 ± 17.72	82.44 ± 17.04	83.72 ± 17.79	0.0596
BMI (kg/m²)	30.95 ± 5.51	30.58 ± 5.36	31.23 ± 5.71	0.0548
Waist circumference (cm)	98.91 ± 12.32	98.71 ± 10.77	106.75 ± 13.52	0.0001***
Hip circumference (cm)	109.18 ± 11.53	107.53 ± 12.05	111.31 ± 11.95	0.0418*
Waist-to-hip ratio	0.91 ± 0.08	0.92 ± 0.08	0.96 ± 0.06	0.0163*
Body fat (%)	32.60 ± 9.27	32.86 ± 10.10	34.33 ± 9.18	0.0622
Body fat mass (kg)	26.94 ± 11.62	27.30 ± 11.76	28.80 ± 12.04	0.0221*
Muscle mass (kg)	52.73 ± 11.69	51.74 ± 11.70	51.97 ± 11.31	0.0910

^*a*^ *P* values were calculated using the Friedman test for repeated measures. The significance levels were defined as follows: *P < 0.05, **P < 0.01, and ***P < 0.001.

^*b*^ Values are reported as mean ± SD.

### Microbial diversity and community structure

**Fig 1:**
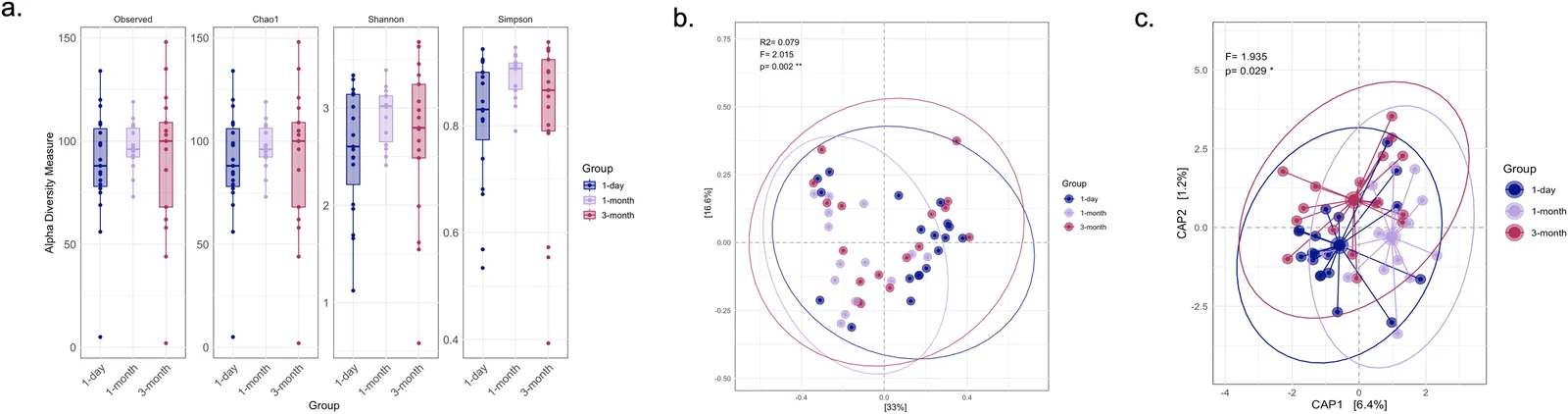
Genus-level alpha and beta diversity of the gut microbiome across Ramadan fasting time points (day 1, 1-month, and 3 months) in prediabetic individuals. (**a**) Alpha diversity metrics (Observed richness, Chao1, Shannon, Simpson). Overall differences across time points were assessed using Friedman repeated-measures tests, followed by paired Wilcoxon post hoc comparisons with false discovery rate (FDR) correction. (**b**) Principal coordinates analysis (PCoA) of Bray-Curtis dissimilarities illustrating genus-level beta diversity. Group differences were tested using PERMANOVA with permutations constrained within individuals. (**c**) Canonical analysis of principal coordinates (CAP) showing time point-associated community separation; ellipses represent 95% confidence intervals.

Genus level alpha diversity exhibited modest but time-dependent variation across Ramadan fasting intervals (Fig. 1a; Tables S1.1 and S1.2). Overall longitudinal comparisons using Friedman repeated-measures tests did not reveal statistically significant changes in richness metrics, including Observed richness (*χ*² = 3.96, df = 2, *P* = 0.138) and Chao1 (*χ*² = 3.96, df = 2, *P* = 0.138). In contrast, Simpson diversity showed a significant overall temporal effect (*χ*² = 8.14, df = 2, *P* = 0.017), with Shannon diversity displaying a similar but non-significant trend (*χ*² = 5.14, df = 2, *P* = 0.076).

Furthermore, post-hoc tests showed that Shannon and Simpson diversities were significantly higher during the 1-month fasting period compared to the baseline (FDR-adjusted *P* = 0.0208 and 0.0095, respectively), while no significant differences were detected for the 3-month fasting period compared to the other groups, indicating a partial reversion to the baseline.

Beta diversity analyses based on Bray-Curtis dissimilarities demonstrated a significant effect of fasting interval on overall community composition (Fig. 1b; Tables S1.3 and S1.4). PERMANOVA analysis showed a significant temporal trend (pseudo-*F* = 2.02, *R*² = 0.079, *P* = 0.002), and the dissimilarities between 1 day and 1-month (FDR-adjusted *P* = 0.006) and 1-month and 3 months (FDR-adjusted *P* = 0.0105) were significant, while the dissimilarities between 1 day and 3 months were not significant. Importantly, homogeneity of multivariate dispersions did not differ across fasting intervals (betadisper, *F* = 0.5235, *P* = 0.5959), indicating that these differences were not driven by variation in within-group dispersion. This finding was also supported by the Canonical Analysis of Principal Coordinates (CAP) method, which showed significant group separations driven by the first axis during the 1-month fasting period (Fig. 1c; Table S1.5).

### Taxonomic composition and temporal dynamics

At the phylum level, the gut microbiota was consistently dominated by Firmicutes and Bacteroidota across all time points. Bacteroidota predominated at baseline, but by 1-month, there was a marked increase in Firmicutes, leading to a transient rise in the Firmicutes-to-Bacteroidota ratio. By 3 months, this ratio partially normalized. Other phyla, including Proteobacteria, Actinobacteriota, and Verrucomicrobiota, remained low in abundance and relatively stable over time (Fig. S3).

**Fig 2:**
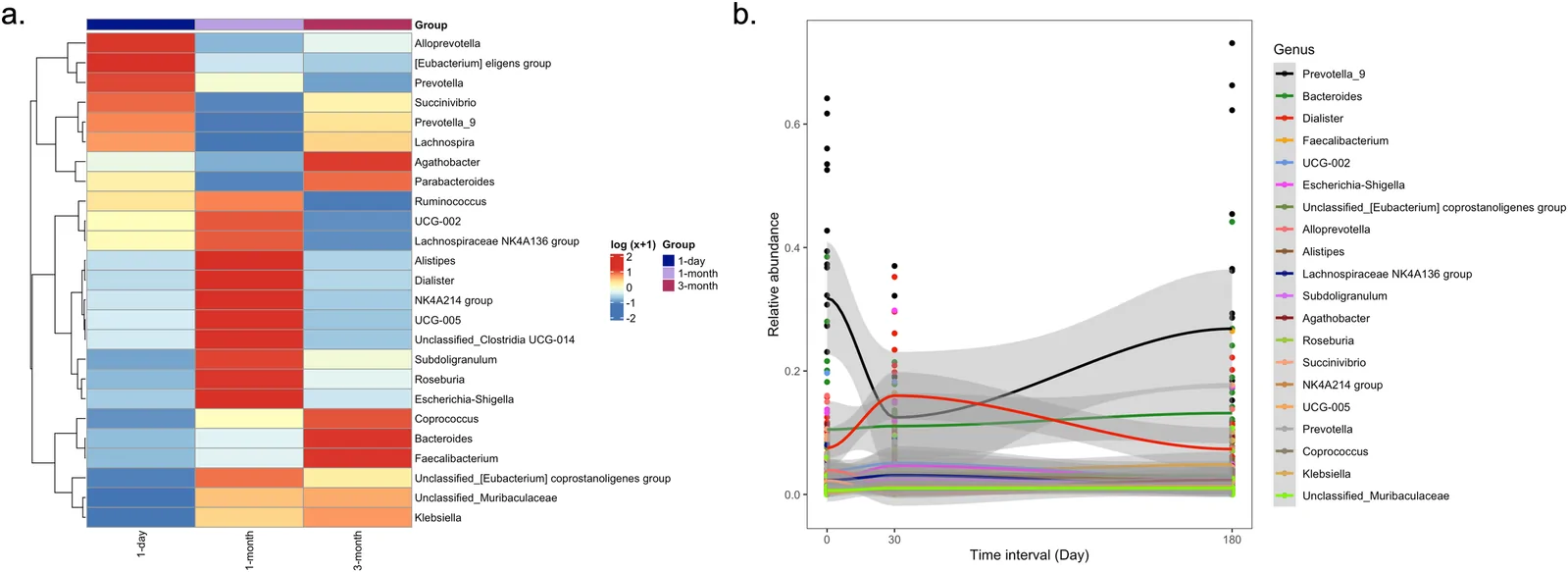
Temporal dynamics of gut microbiota composition across timepoints (1-day, 1-month, and 3-month). (**a**) Heatmap of the 25 most abundant genera, clustered using complete-linkage hierarchical clustering, illustrating time-dependent compositional patterns. Values are log(*x* + 1) transformed and scaled by genus. (**b**) Longitudinal trajectories of the 20 major genera, highlighting dynamic shifts in relative abundances across study intervals.

At genus level, heatmap analysis of the 25 most abundant genera revealed clear interval-specific signatures (Fig. 2a). Baseline samples were characterized by higher relative abundance of *Prevotella_9* and *Bacteroides*. The end-of-Ramadan microbiome showed the enrichment of facultative anaerobic and bile-tolerant genera, including *Dialister, Escherichia-Shigella,* and *Alistipes*. By 3 months post-fasting, increased relative abundance of butyrate-producing genera such as *Faecalibacterium, Agathobacter*, and *Roseburia* was observed.

Longitudinal LOESS trajectories further illustrated staggered temporal dynamics among dominant genera (Fig. 2b). *Prevotella_9* declined sharply at 1-month and partially recovered by 3 months, whereas *Faecalibacterium, Agathobacter*, and *Roseburia* increased progressively over time. *Bacteroides* remained relatively stable with a modest upward trend.

### Microbiome-metabolic associations

**Fig 3:**
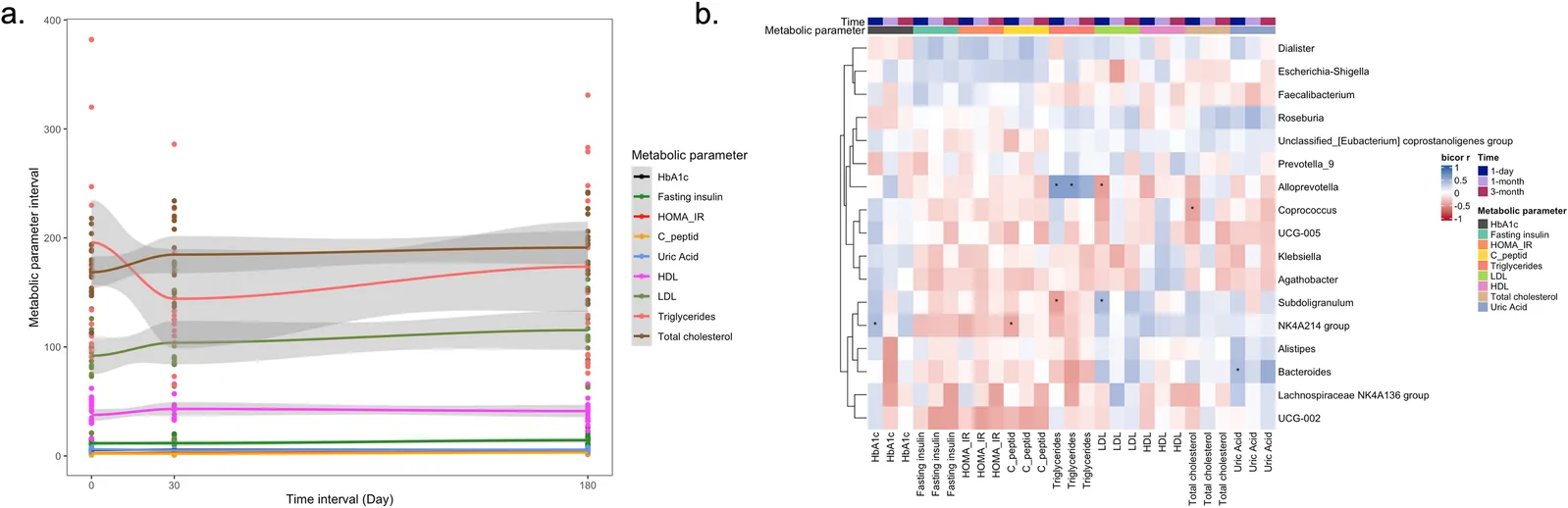
Associations between gut microbiota and metabolic parameters across Ramadan fasting intervals. (**a**) Longitudinal dynamics of key metabolic parameters across study groups (1-day, 1-month, 3-month). Solid lines represent LOESS (locally estimated scatterplot smoothing) regression fits, and shaded areas indicate the 95% confidence intervals. Values are normalized and centered by row. (**b**) Heatmap showing biweight midcorrelation (bicor) coefficients between the relative abundances of the top 20 gut bacterial genera and host metabolic parameters across fasting intervals (1-day, 1-month, and 3-month). Correlation strength and direction are indicated by color gradients, with warm tones representing negative and cool tones positive associations. Asterisks denote statistically significant associations defined as |*r*| ≥ 0.35 with false discovery rate (FDR)-adjusted *P* < 0.10 within each time interval.

Metabolic responses to Ramadan fasting were heterogeneous (Fig. 3a). HbA1c levels showed a slight numerical increase from baseline (5.52 ± 0.30) to the end of Ramadan (5.63 ± 0.28) though this trend did not reach statistical significance (*P* = 0.2430; Table S2). HOMA-IR increased progressively, reaching higher values at 3 months compared with baseline (Wilcoxon signed-rank (paired) 1-day (2.46 ± 0.72) vs 3-month (3.56 ± 1.49) *P* = 0.032; Table 1). C-peptide increased significantly over time, whereas fasting insulin showed a non-significant upward trend. Triglycerides decreased at 1-month and partially rebounded by 3 months, while LDL, HDL, and total cholesterol did not show significant longitudinal changes (Table S2; Table 1).

Correlation analysis using biweight midcorrelation identified time-dependent associations between microbial genera and metabolic parameters (Fig. 3b). At baseline and at the end of Ramadan, several genera showed associations with lipid and glycemic indices, including relationships involving *Alloprevotella, Subdoligranulum*, and triglyceride or LDL levels. In contrast, few significant microbiome-metabolic correlations were detected at the 3-month time point.

### Differentially abundant taxa and indicator species

**Fig 4:**
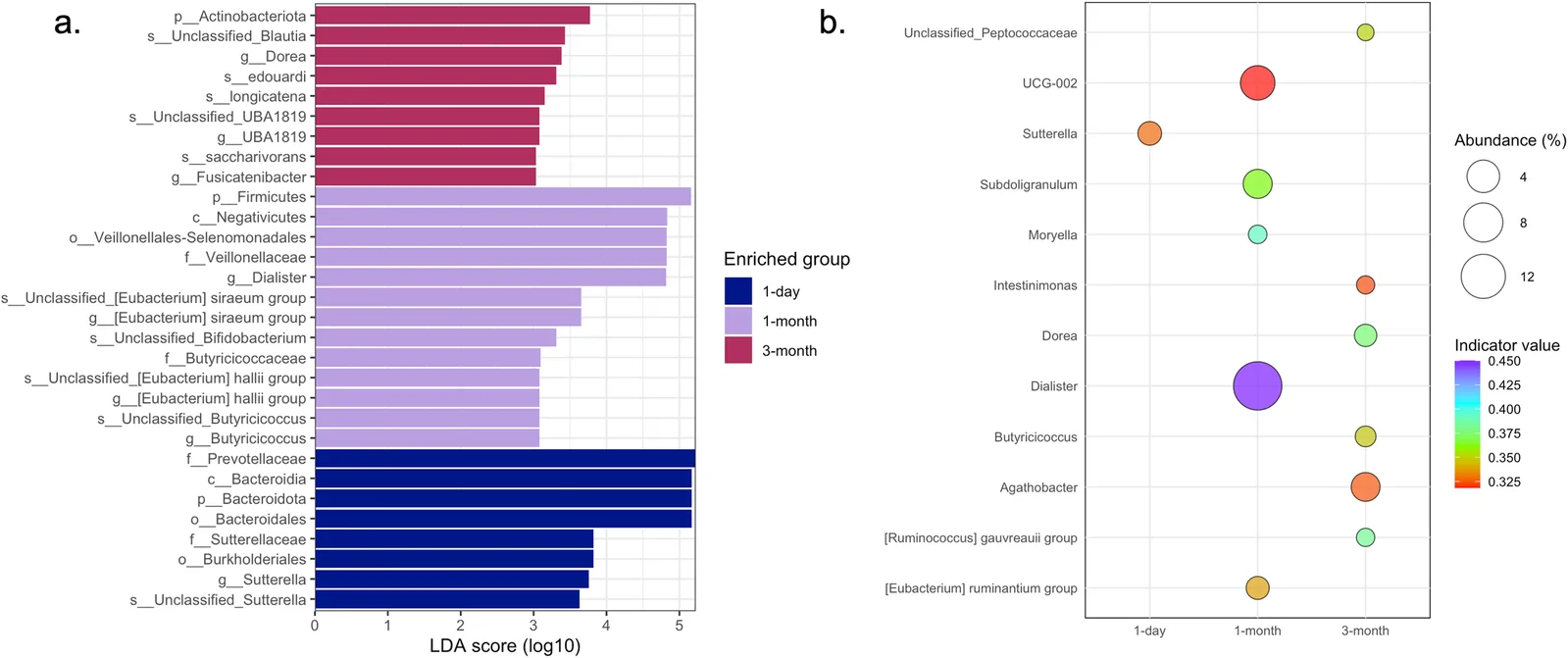
Taxa associated with Ramadan fasting time points. (**a**) Linear discrimination analysis (LDA) effect size (LEfSe) analysis identifying taxa differentially enriched across time points (LDA score > 3.0, *P* < 0.05). Taxonomic ranks are indicated by prefixes (p, phylum; c, class; o, order; f, family; g, genus; s, species). (**b**) Indicator species analysis showing genera significantly associated with specific time points (IndVal > 0.30, *P* < 0.05). Bubble size reflects mean relative abundance, and color indicates indicator value.

Differential abundance analyses were used to determine which taxa were associated with the changes in community composition over time (Fig. 4a). LEfSe analysis revealed enrichment of *Bacteroides, Prevotella_9,* and *Dialister* at baseline; SCFA-associated genera (*Faecalibacterium, Subdoligranulum,* and *Roseburia*) at 1-month post-intervention; and butyrate-associated genera (*Agathobacter, Butyricicoccus,* and *Dorea*) at 3 months (LDA > 3.0, *P* < 0.05). The results were consistent with the indicator species analysis, which revealed taxa significantly associated with a given time point (IndVal > 0.3, *P* < 0.05; Fig. 4b).

The core microbiome composition revealed that dominant genera were present across all sampling intervals (Fig. S3), implying that Ramadan fasting did not replace dominant core genera, but altered their relative abundance. This finding is consistent with the observation that the mean relative abundance profiles revealed a gradual increase in the relative abundance of Bacteroides and SCFA-associated genera between baseline and three months (Fig. S5).

### Multivariable association with host metadata

**Fig 5:**
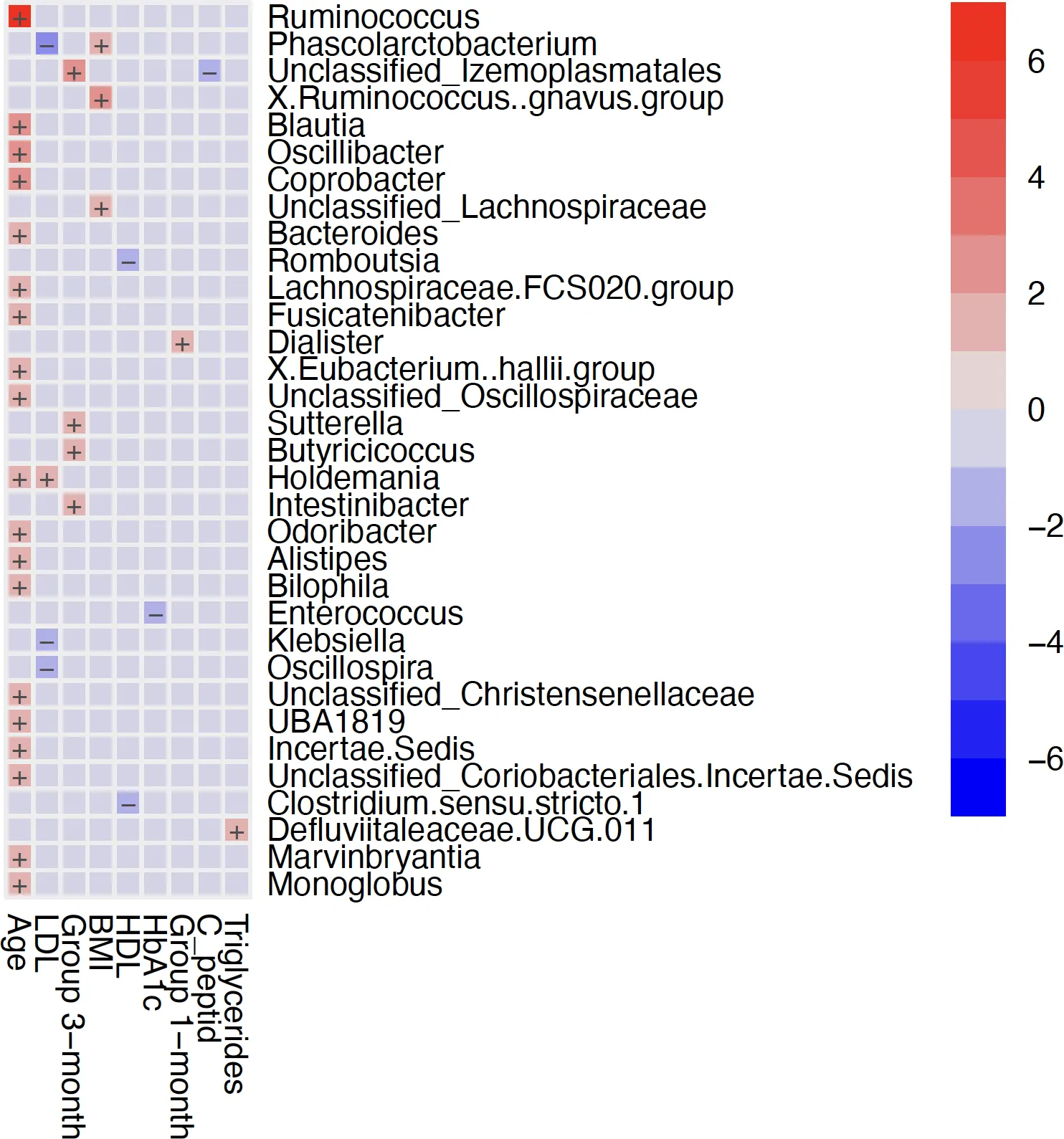
Multivariable associations between gut microbial genera and host metadata identified by MaAsLin2. Heatmap illustrating significant associations between microbial taxa and clinical parameters across Ramadan fasting intervals (*q* < 0.25). Effect sizes were calculated as −log(*q*-value) × SIGN(coefficient), where positive values (red) indicate positive associations and negative values (blue) indicate inverse associations. The intensity of the color represents the magnitude of the association. Node sizes reflect the strength and connectivity of features within the model.

MaAsLin2 modeling identified associations between microbial taxa and host metadata (*q* < 0.25; Fig. 5). Age was positively associated with multiple genera, including *Ruminococcus, Blautia, Oscillibacter, Odoribacter, Alistipes,* and *Bilophila*. BMI showed positive associations with the *Ruminococcus gnavus group, Phascolarctobacterium*, and unclassified *Lachnospiraceae*. Several genera exhibited associations with lipid parameters, including inverse relationships between *Phascolarctobacterium* or *Klebsiella* and LDL cholesterol

Timepoint-specific associations were also observed, including enrichment of *Dialister* at 1-month and *Sutterella, Butyricicoccus*, and *Intestinibacter* at 3 months. Unclassified *Izemoplasmatales* was more abundant at 3 months and inversely associated with C-peptide.

### Predicted functional pathway dynamics

PICRUSt2-based functional inference revealed distinct temporal shifts in predicted microbial metabolic potential across the Ramadan fasting timeline (Fig. S8). At the pre-Ramadan baseline (day 1), microbiomes were enriched for pathways related to carbohydrate and nucleotide metabolism, including thiazole biosynthesis (LDA = 2.8), pyrimidine degradation (LDA = 2.5), and the TCA cycle VII (LDA = 2.3).

At the end of Ramadan, predicted functions shifted toward SCFA-related fermentation pathways and amino acid biosynthesis, including methionine, isoleucine, and lysine pathways, alongside increased representation of purine and pyrimidine degradation pathways. At 3 months post-fasting, pathways related to fatty acid elongation, monounsaturated fatty acid biosynthesis, and butanoate fermentation were enriched (Fig. S8).

## DISCUSSION

This longitudinal study examined how Ramadan fasting affects gut microbiome structure, predicted functional capacity, and metabolic outcomes in adults with prediabetes. Our findings support the first part of this hypothesis, demonstrating phase-specific microbial compositional shifts together with changes in predicted functional profiles, but they do not demonstrate consistent short-term metabolic improvement (24, 25). Instead, several anthropometric and insulin resistance indices worsened over time, highlighting the complexity of fasting responses in a metabolically vulnerable population (26).

Notably, in our cohort, these microbial trajectories occurred in the context of stable HbA1c and fasting glucose, rising HOMA-IR, and increased waist circumference and fat mass. C-peptide was measured as a complementary marker of endogenous insulin secretion and β-cell function; unlike insulin, C-peptide is not subject to hepatic first-pass metabolism, providing a more stable indicator of pancreatic insulin production. Together, progressive increases in both HOMA-IR and C-peptide over the study period indicate worsening insulin resistance with compensatory β-cell hypersecretion. The presence of taxa often considered metabolically favorable, without parallel improvement in clinical readouts, suggests that prediabetic physiology and real-world behavior can blunt or override potential microbiome-mediated benefits (27).

Microbiota-metabolic associations were also time-dependent. During baseline and the fasting interval, several taxa showed coherent relationships with lipid and glycemic markers, whereas these associations were weaker or less consistent three months post-Ramadan. For example, SCFA-producing genera such as *Roseburia*, *Agathobacter*, and *Faecalibacterium* aligned with more favorable lipid parameters during the fasting period (28, 29). In contrast, taxa commonly linked to opportunistic and pro-inflammatory infections, including *Escherichia-Shigella*, *Bacteroides*, and *Succinivibrio* aligned with higher insulin resistance, triglycerides, and glycemic markers, especially at the end of Ramadan (29). These findings are consistent with prior work linking *Escherichia coli/Shigella* overgrowth and certain *Bacteroides* species to dysmetabolism and insulin resistance in high-risk populations (30, 31). Despite phase-specific microbiota-metabolic associations, these responses did not translate into consistent clinical improvement, indicating that microbial responsiveness alone was insufficient to overcome underlying metabolic dysregulation.

The inclusion of an age- and sex-matched healthy reference cohort further demonstrated that prediabetic participants harbored a compositionally distinct gut microbiome at baseline prior to Ramadan fasting. These findings suggest that fasting-associated microbial remodeling occurred within an already altered microbial ecosystem characteristic of impaired metabolic health. Thus, although Ramadan fasting was associated with enrichment of selected SCFA-associated taxa and increased microbial diversity, these microbiome changes were not accompanied by measurable metabolic improvement in this cohort. One possible explanation is that reduced metabolic flexibility and underlying host dysregulation associated with prediabetes may limit the extent to which microbiome-associated changes translate into short-term metabolic benefit.

Additional multivariate and network-based analyses supported a temporally phased organization of microbiome responses during and after Ramadan fasting, consistent with coordinated, interval-specific community reorganization (Fig. 5; Fig. S11).

When considered in the context of Ramadan fasting studies in diabetes and high-risk cardiometabolic populations, the metabolic profile observed here is not anomalous but rather lies the “neutral to adverse” end of a heterogeneous spectrum. Several observational and interventional studies in people with type 2 diabetes have reported modest reductions in glucose, HbA1c, blood pressure, and lipids after Ramadan, particularly when patients receive structured education and medication adjustment (32–36). In contrast, other cohorts have shown either no meaningful change in insulin sensitivity or even deterioration of glycemic control, with Ramadan fasting associated with higher post Ramadan HbA1c or adverse lipid shifts, especially when diet quality is poor, or support is limited (37–40). Systematic reviews in high-risk groups, such as individuals with chronic kidney disease, similarly emphasize variability in HbA1c and lipid responses and stress the importance of diet composition and clinical supervision (41). Our findings align more closely with these neutral or adverse patterns, suggesting that in prediabetes, Ramadan fasting is not inherently beneficial and may even exacerbate insulin resistance and central adiposity under real-world eating behaviors.

The discrepancy between microbial “improvement” and metabolic stagnation or worsening likely reflects a combination of behavioral and host factors. In people with diabetes, continuous glucose monitoring studies frequently show pronounced postprandial glucose excursions after evening and pre-dawn meals during Ramadan, which may worsen overall glycemic dynamics despite daytime caloric restriction in some settings (42, 43). The net metabolic impact of Ramadan fasting appears to be driven by compensatory eating behaviors, circadian disruption, and limited metabolic flexibility rather than microbiome plasticity alone.

Functional predictions add another layer to this picture. We observed a staged shift from carbohydrate- and nucleotide-related pathways at baseline to SCFA and amino acid biosynthesis during Ramadan and then to lipid- associated pathways at 3 months. Such temporal reprogramming has been reported in other fasting and time-restricted feeding studies and is often interpreted as microbial adaptation to altered nutrient availability (44, 45). In our cohort, these changes did not translate into better glycemic control or improved lipid profile.

### Conclusion

Taken together, Ramadan fasting was associated with temporal remodeling of the gut microbiome in prediabetic adults, but these changes did not lead to uniform metabolic improvement. One possible interpretation is that, in prediabetes, microbiome-associated changes occurring during Ramadan fasting may be influenced by compensatory eating behaviors, circadian disruption, and limited metabolic flexibility.

This study has several strengths, including longitudinal sampling across three clinically relevant time points, integration of taxonomic, predicted functional, and network-based analyses, and a focus on a prediabetic cohort rather than metabolically healthy volunteers. Nevertheless, several limitations should be considered when interpreting the findings. First, the relatively small cohort size (*n* = 17) from a single center limits statistical power for subgroup analyses and may reduce generalizability to broader populations. Second, although technical replicates demonstrated high concordance and all prediabetic samples were processed within a single sequencing batch, broader technical replication across multiple participants would provide a more comprehensive assessment of analytical reproducibility. Third, dietary intake was not formally quantified. Consequently, the effects of fasting itself cannot be fully separated from concurrent Ramadan-associated changes in meal timing, dietary composition, and circadian patterns. In addition, the healthy reference cohort was incorporated to provide baseline biological context for the prediabetic microbiome but was not sampled longitudinally during Ramadan. Therefore, comparisons involving the healthy cohort should be interpreted as cross-sectional reference analyses rather than assessments of differential fasting responses. Finally, predicted functional profiles were inferred computationally using PICRUSt2 and should be considered hypotheses regarding metabolic potential rather than direct measurements of microbial activity. These limitations restrict causal inference and mechanistic interpretation of the observed associations. Future studies incorporating larger multi-center cohorts, prospective dietary assessment, longitudinal healthy-control sampling, and direct metagenomic, metabolomic, and functional measurements will help further clarify the biological and clinical significance of Ramadan-associated microbiome remodeling in adults with prediabetes.

## Data Availability

Raw sequencing reads that support the findings of this study have been deposited in the NCBI BioProject database under the following accession numbers: PRJNA1329139.

## AFTER EPUB

*[This article was published on 3 August 2026 with an error in the affiliations. The affiliations were corrected in the current version, posted on 5 August 2026.]*

## SUPPLEMENTAL MATERIAL

File S1 spectrum.00742-26-s0001.xlsx Supplemental analysis.

Supplemental materials spectrum.00742-26-s0002.docx Supplemental tables, figures, and text.

Supplemental materials and methods spectrum.00742-26-s0003.docx Supplemental materials and methods

ASM does not own the copyrights to Supplemental Material that may be linked to, or accessed through, an article. The authors have granted ASM a non-exclusive, world-wide license to publish the Supplemental Material files. Please contact the corresponding author directly for reuse.
