# Supplementary material for: Ramadan fasting induces temporal gut microbiome remodeling without metabolic improvement in adults with prediabetes
Source: Microbiol Spectr. 2026 Aug 3;14(9):e00742-26. doi: 10.1128/spectrum.00742-26 (PMC13532256; doi:10.1128/spectrum.00742-26)
Supplement: Supplementary file 1 [file spectrum.00742-26-s0002.docx]

**Supplementary File_S1_Supporting Document**

**Ramadan Fasting Is Associated with Temporal Gut Microbiome Remodeling Without Metabolic Improvement in Adults with Prediabetes**

Ihsan Boyaci^1^, Busranur Delice^2^, Fatma Koc^2&^, Suleyman Yildirim^2,3^*

^1^Istanbul Medipol University, Vatan Clinic, Istanbul, 34214, Türkiye.

^2^Department of Medical Microbiology, Istanbul Medipol University International School of Medicine, Istanbul, Türkiye.

^3^Regenerative and Restorative Medicine Research Center, REMER, Istanbul Medipol University & Research Institute for Health Sciences and Technologies (SABITA), Istanbul Medipol University, Istanbul, Türkiye.

**^*^**Corresponding author

E-mail: [suleymanyildirim@medipol.edu.tr](mailto:suleymanyildirim@medipol.edu.tr)

^&^Current Address: APC Microbiome Ireland, University College Cork, Ireland

## **Table S1.1.** Genus-level alpha diversity across fasting intervals (1-day, 1-month, and 3-month) assessed using Observed, Chao1, Shannon, and Simpson indices. Overall longitudinal differences were evaluated using Friedman repeated-measures tests.

| Measure Types | Friedman chi-squared | df | p-value |
| --- | --- | --- | --- |
| Observed | 3.9636 | 2 | 0.1378 |
| Chao1 | 3.9636 | 2 | 0.1378 |
| Shannon | 5.1428 | 2 | 0.0764 |
| Simpson | 8.1428 | 2 | 0.0170** |

*Signif. codes: 0 ‘***’ 0.001 ‘**’ 0.01 ‘*’ 0.05 ‘.’ 0.1 ‘ ’ 1*

## **Table S1.2.** Pairwise comparisons of genus-level alpha diversity indices between fasting intervals using paired Wilcoxon signed-rank tests with false discovery rate (FDR) correction.

| Comparison | Observed (p) | FDR-adjusted p | Chao1 (p) | FDR-adjusted p | Shannon (p) | FDR-adjusted p | Simpson (p) | FDR-adjusted p |
| --- | --- | --- | --- | --- | --- | --- | --- | --- |
| 1-day – 1-month | 0.0833 | 0.2500 | 0.0834 | 0.2500 | 0.0069** | 0.0208* | 0.0031** | 0.0095** |
| 1-day – 3-month | 0.3149 | 0.4724 | 0.3149 | 0.4724 | 0.4144 | 0.4145 | 0.6154 | 0.6155 |
| 1-month – 3-month | 0.8508 | 0.8506 | 0.8506 | 0.8506 | 0.4144 | 0.4144 | 0.2092 | 0.3139 |

*Signif. codes: 0 ‘***’ 0.001 ‘**’ 0.01 ‘*’ 0.05 ‘.’ 0.1 ‘ ’ 1*

**Table S1.3.** Genus-level beta diversity comparisons across fasting intervals (1-day, 1-month, and 3-month) based on Bray-Curtis dissimilarities, assessed using PERMANOVA with permutations constrained within individuals to account for repeated measurements.

| Source | Df | SumOfSqs | R² | F | p-value |
| --- | --- | --- | --- | --- | --- |
| Group | 2 | 0.6604 | 0.0789 | 2.0154 | 0.0020** |
| Residual | 47 | 7.7006 | 0.9210 |  |  |
| Total | 49 | 8.3610 | 1.0000 |  |  |

*Number of permutations: 999. Signif. codes: 0 ‘***’ 0.001 ‘**’ 0.01 ‘*’ 0.05 ‘.’ 0.1 ‘ ’ 1*

## **Table S1.4.** Pairwise PERMANOVA comparisons of genus-level beta diversity between fasting intervals based on Bray-Curtis dissimilarities, performed using subject-blocked permutations and false discovery rate (FDR) correction.

| Comparison | F | R^2^ | p-value | FDR-adjusted p |
| --- | --- | --- | --- | --- |
| 1-day – 1-month | 3.4915 | 0.1012 | 0.002** | 0.006** |
| 1-day – 3-month | 0.8289 | 0.0238 | 0.12 | 0.12 |
| 1-month – 3-month | 1.9000 | 0.0614 | 0.007** | 0.0105** |

*Number of permutations: 999. Signif. codes: 0 ‘***’ 0.001 ‘**’ 0.01 ‘*’ 0.05 ‘.’ 0.1 ‘ ’ 1*

**Table S1.5. Constrained analysis of principal coordinates (CAP) on Bray–Curtis distances.**

| **Overall test:** F = 1.935, p = 0.029* | |
| --- | --- |
| **One-df test:** | **Axis test:** |
| - 1-month: F = 3.0237, p = 0.010** | - CAP1: F = 3.2449, p = 0.024* |
| - 3-month: F = 0.8462, p = 0.554 | - CAP2: F = 0.6250, p = 0.798 |

*Number of permutations: 999. Signif. codes: 0 ‘***’ 0.001 ‘**’ 0.01 ‘*’ 0.05 ‘.’ 0.1 ‘ ’ 1*

**Table S1.6. Pairwise Bray-Curtis dissimilarity matrix of technical replicates (triplicates) from a single participant at the 1-month time point.**

Values represent the dissimilarity between community profiles, where 0 indicates identical composition and 1 indicates complete dissimilarity. The low mean dissimilarity (0.056) indicates high technical reproducibility.

| Technical replicates | GU54M6R1 (distance) | GU54M6R2 (distance) | GU54M6R3 (distance) |
| --- | --- | --- | --- |
| GU54M6R1 | 0.0000 | 0.0725 | 0.0713 |
| GU54M6R2 | 0.0725 | 0.0000 | 0.0251 |
| GU54M6R3 | 0.0713 | 0.0251 | 0.0000 |

**Table S1.7. PERMANOVA results for HC vs 1-day (Group, Age, Sex; Type III).**

|  | Df | Sum Of Sqs | R^2^ | F | Pr (>F) |
| --- | --- | --- | --- | --- | --- |
| Group | 1 | 0.9237 | 0.1340 | 4.7294 | 8 |
| Age | 1 | 0.1179 | 0.0171 | 0.6039 | 643 |
| Sex | 1 | 0.1355 | 0.0196 | 0.6940 | 573 |
| Residual | 29 | 5.6643 | 0.8217 |  |  |
| Total | 32 | 6.8928 | 1 |  |  |

**Table S1.8. PERMANOVA results for all four groups.**

|  | Df | Sum Of Sqs | R^2^ | F | Pr (>F) |
| --- | --- | --- | --- | --- | --- |
| Group | 3 | 2.2416 | 0.1743 | 4.2755 | 1 |
| Age | 1 | 0.2766 | 0.0215 | 1.5832 | 148 |
| Sex | 1 | 0.2375 | 0.0184 | 1.3591 | 208 |
| Residual | 57 | 9.9615 | 0.7746 |  |  |
| Total | 62 | 12.859 | 1 |  |  |

**Table S1.9. Wilcoxon test results**

HC vs 1-day for all alpha diversity metrics.

| Metric | group1 | group2 | p | p.adj | p.adj.signif |
| --- | --- | --- | --- | --- | --- |
| Chao1 | HC | 1-day | 0.00000104 | 0.00000138 | **** |
| Observed | HC | 1-day | 0.00000104 | 0.00000138 | **** |
| Shannon | HC | 1-day | 0.000000466 | 0.00000138 | **** |
| Simpson | HC | 1-day | 0.0000369 | 0.00003690 | **** |

**Table S1.10. Dunn post-hoc results all pairwise comparisons across four groups.**

| Metric | group1 | group2 | statistic | p | p.adj | p.adj.signif |
| --- | --- | --- | --- | --- | --- | --- |
| Observed | HC | 1-day | 4.4509 | 0.0000 | 0.0000 | **** |
| Observed | HC | 1-month | 4.7527 | 0.0000 | 0.0000 | **** |
| Observed | HC | 3-month | 5.4302 | 0.0000 | 0.0000 | **** |
| Observed | 1-day | 1-month | 0.4507 | 0.6522 | 0.6521 | ns |
| Observed | 1-day | 3-month | 0.9648 | 0.3346 | 0.5019 | ns |
| Observed | 1-month | 3-month | 0.4813 | 0.6302 | 0.6521 | ns |
| Shannon | HC | 1-day | 3.8620 | 0.0001 | 0.0002 | *** |
| Shannon | HC | 1-month | 4.8434 | 0.0000 | 0.0000 | **** |
| Shannon | HC | 3-month | 4.9877 | 0.0000 | 0.0000 | **** |
| Shannon | 1-day | 1-month | 1.1007 | 0.2710 | 0.3252 | ns |
| Shannon | 1-day | 3-month | 1.1090 | 0.2674 | 0.3252 | ns |
| Shannon | 1-month | 3-month | -0.02928 | 0.9766 | 0.9766 | ns |
| Simpson | HC | 1-day | 3.3121 | 0.0009 | 0.0018 | ** |
| Simpson | HC | 1-month | 4.9133 | 0.0000 | 0.0000 | **** |
| Simpson | HC | 3-month | 4.3497 | 0.0000 | 0.0000 | **** |
| Simpson | 1-day | 1-month | 1.6930 | 0.0904 | 0.1356 | ns |
| Simpson | 1-day | 3-month | 1.0222 | 0.3066 | 0.3679 | ns |
| Simpson | 1-month | 3-month | -0.7054 | 0.4805 | 0.4805 | ns |

**Table S1.11. Pairwise Bray-Curtis distance matrix for technical replicates.**

|  | GU54M6 | GU54M6R2 | GU54M6R3 |
| --- | --- | --- | --- |
| GU54M6 | 0 | 0.0744 | 0.0725 |
| GU54M6R2 | 0.0744 | 0 | 25 |
| GU54M6R3 | 0.0725 | 25 | 0 |

**Table S1.12.** Baseline demographic characteristics of the Healthy Control (HC) and Prediabetic cohorts.

| Variable | Healthy Controls (HC) (n=17) | Prediabetic Cohort (n=17) | p-value |
| --- | --- | --- | --- |
| Sex (Male / Female) | 7 / 10 | 7 / 10 | 0.999^a^ |
| Age (years), *mean $\pm$ SD* | 49.85 ± 5.24 | $50.24 ± 5.57$ | 0.835^b^ |
| Age Range (years) | 41-63 | 40-65 | - |

*Values are expressed as absolute counts for categorical variables and as mean  standard deviation (SD) for continuous variables. The cohorts are precisely matched for both age and sex criteria to ensure cross-sectional comparability at baseline.*  *Calculated using Fisher's exact test.*  *Calculated using an independent Student's t-test or Mann-Whitney U test.*

**Figure S1. Technical Reproducibility and Beta Diversity. PCoA plot based on Bray-Curtis dissimilarities at the genus level.**

Samples are colored by group: 1-day (dark blue), 1-month (light purple), and 3-month (maroon). Technical replicates (n=3) from a single participant are indicated in bright red and connected by a red polygon to highlight their tight clustering. The high congruence of replicates (mean distance = 0.056) relative to biological variation confirms the precision of the analysis.

**Table S2. Longitudinal changes in metabolic parameters across Ramadan fasting intervals in prediabetic individuals.**

Values are presented as mean ± standard deviation (SD) at day 1 (baseline), 1 month, and 3 months post-fasting. Statistical differences were assessed using the Friedman repeated-measures test, with corresponding χ²-values, degrees of freedom, uncorrected p-values, and FDR-adjusted p-values reported.

| **Metabolic parameters** | **1-day (mean ± std)** | **1-month (mean ± std)** | **3-month (mean ± std)** | **χ²-values** | **Df** | **p**  **uncorrected** | FDR-adjusted p |
| --- | --- | --- | --- | --- | --- | --- | --- |
| **HbA1c** | 5.5211 ± 0.2955 | 5.6286 ± 0.2840 | 5.6118 ± 0.2848 | 3.04 | 2 | 0.2187 | 0.2430 |
| **BMI** | 30.8705 ± 5.2010 | 30.2907 ± 5.6742 | 31.5676 ± 5.7003 | 5.9273 | 2 | 0.0516 | 0.0996 |
| **Fasting insulin** | 11.6158 ± 3.3826 | 11.7379 ± 5.1432 | 14.4247 ± 5.3844 | 5.5714 | 2 | 0.0616 | 0.0996 |
| **HOMA_IR** | 2.4611 ± 0.7221 | 2.7186 ± 1.2211 | 3.5606 ± 1.4885 | 8.1429 | 2 | 0.0170 | 0.0483 |
| **C_peptid** | 2.1289 ± 0.3714 | 2.1021 ± 0.6001 | 3.1494 ± 0.9543 | 18.5818 | 2 | 0.0000 | 0.0009 |
| **Uric Acid** | 6.0105 ± 1.1160 | 5.2000 ± 1.0160 | 5.5824 ± 1.2089 | 5.2857 | 2 | 0.0711 | 0.0996 |
| **HDL** | 37.6842 ± 13.0854 | 43.1429 ± 9.2807 | 41.1765 ± 11.6256 | 5.0588 | 2 | 0.0797 | 0.0996 |
| **LDL** | 91.8947 ± 38.9842 | 104.0000 ± 41.7133 | 115.4118 ± 30.0688 | 12.3333 | 2 | 0.0020 | 0.0104 |
| **Triglycerides** | 196.1053 ± 101.3212 | 144.1429 ± 62.5839 | 173.6471 ± 79.9820 | 1 | 2 | 0.6065 | 0.6065 |
| **Total cholesterol** | 168.5263 ± 31.3517 | 184.7143 ± 32.2310 | 191.1765 ± 31.2675 | 7.8909 | 2 | 0.0193 | 0.0483 |

**Figure S2. Linear regression models were used to analyze the Simpson index of alpha diversity across Ramadan fasting intervals: (a) 1-day vs. 1-month, (b) 1-day vs. 3-month, and (c) 1-month vs. 3-month.**

The fitted lines represent local regression correction curves, with shaded areas indicating the 95% confidence intervals.

**Figure S3. Temporal dynamics of gut microbiota composition across fasting intervals (1-day, 1-month, and 3-month) in prediabetic individuals during Ramadan.**

Stacked bar plots depicting the relative abundances of the top 10 bacterial phyla across groups.

**Figure S4. Heatmap of core microbiome structures at the genus level for the study time intervals: (a) 1-day, (b) 1-month, and (c) 3-month.**

Heatmap of the core microbiome structure of regions at genus level as a function of the abundance threshold for taxa with prevalence above 0.5. The x-axis represents the detection thresholds (expressed as relative abundance) from lower (left) to higher (right) values. Color shading reflects the prevalence of each genus in the samples at each abundance level.

**Figure S5. To investigate the dynamics of bacterial taxa between Ramadan fasting-based time intervals.**

Mean relative abundance of genera across time points. Genera with <0.9% mean relative abundance were omitted for clarity.

**Figure S6. The association between metadata and the gut microbiome at the genus level was analyzed using linear mixed models implemented in MaAsLin2.**

This approach comprehensively evaluated the relationships between various metadata variables and microbial abundances (a), accounting for possible confounding factors (b) and random effects.

**Figure S7. The association between the gut microbiome at the genus level and, microbial parameters was analyzed using linear mixed models implemented in MaAsLin2.**

This approach comprehensively evaluated the relationships between various metabolic parameters and microbial abundances, accounting for possible confounding factors and random effects.

**Figure S8. Functional pathways and enzymes predicted by PICRUSt2 that differentiate in study groups.**

Heatmap of top 20 mean abundant predicted pathway (a) and, enzymes (b) which were clustered based on complete-linkage hierarchical clustering. Histogram of the LDA scores computed for pathway (c) and, enzymes (d) differentially abundant between time intervals. Listed are predicted pathways with LDA scores (log10) > 2 and p < 0.05, and predicted enzymes with LDA scores (log10) > 2.5 and p < 0.05.

**Figure S9. Functional pathways and enzymes predicted by PICRUSt2 that differentiate in study groups.**

Analysis of indicator species in study time intervals. Differentially enriched predicted pathway (a) and, enzymes (b) indicator species in time periods on based ramadan fasting. The only indicator values shown are those that are significant (IndVal >0.3, p < 0.05). The color scale bar displays the indicator value for each pathway or enzymes, and the size of the bubble symbol is proportionate to the mean relative abundance of indicator species.

**Figure S10. Multiple biweight midcorrelations between predicted pathways, bacteria genus and, metabolic parameters in the study time intervals.**

Heatmaps of multiple biweight midcorrelations (bicor) analysis showing the relative abundances of the top 20 abundant pathways and genera (a) and the top 20 abundant pathways and metabolic parameters (b).Indicator: the correlation coefficients; warm tone represents negative correlation; cold tone represents positive correlation; Only significant correlations *(r=±0.35, p<0.05).

**Figure S11. Multi-omics integration of gut microbiome and metabolic features across Ramadan fasting intervals using MintTea**

(a) Network analysis of multi-omics modules identified by sparse generalized canonical correlation analysis (sGCCA). Nodes represent features (microbial taxa or metabolic parameters), node size reflects feature weight, and colors denote module membership. Edges represent stable feature co-occurrence across >80% of subsampling iterations and are colored by time-point comparison (1-day vs 1-month: blue; 1, 1-day vs 3-month: pink; 2, 1-month vs 3-month: lilac; 3).

(b) Summary characteristics of consensus modules, including the number of features per module, cross-omics average correlation, and classification performance (AUC). Modules composed of a single omic layer were excluded.

**Figure S12. Batch Effect Validation (ConQuR)**

Principal Coordinates Analysis (PCoA) based on Bray-Curtis dissimilarity at genus level, displayed before (left) and after (right) ConQuR batch correction. Points are colored by study group (HC, 1-day, 1-month, 3-month) and shaped by sequencing batch (circle = Prediabetic Batch; triangle = HC Batch). Dashed ellipses represent 95% confidence intervals by batch. Batch R² before correction = 0; after correction = 0, confirming that the dominant source of compositional variance was biological group membership (R² = 0.029) rather than technical batch. The absence of measurable batch variance reflects perfect collinearity between Batch and Group in this study design.

**Figure S13. Alpha Diversity Across Groups**

Alpha diversity indices (Observed richness, Shannon index, Simpson index) across healthy controls (HC, n=17) and prediabetic participants at baseline (1-day, n=17), 1-month (n=14), and 3-month (n=17) time points. Boxes show median and interquartile range; individual data points are overlaid. All three-diversity metrics were significantly higher in HC compared to all prediabetic time points (Dunn post-hoc test with BH correction, p < 0.001 for all HC comparisons). No significant differences were observed between prediabetic time points (all p > 0.05), indicating that Ramadan fasting does not restore microbiome diversity to healthy control levels.

**Figure S14. Beta Diversity (Bray-Curtis PCoA)**

(A) Principal Coordinates Analysis (PCoA) based on Bray-Curtis dissimilarity comparing healthy controls (HC, n=17) and prediabetic participants at baseline (1-day, n=17). Ellipses represent 95% confidence intervals. PERMANOVA (999 permutations, Type III, covariates: age and sex) demonstrated significant compositional separation between groups (R² = 0.134, F = 4.729, p = 0.008). Age and sex were non-significant covariates (p > 0.5 for both).

(B) PCoA including all four study groups (HC, 1-day, 1-month, 3-month). PERMANOVA revealed significant overall compositional differences (R² = 0.174, F = 4.276, p = 0.001), with the Group effect accounting for 17.4% of total compositional variance.

**Figure S15. Technical Replicate Clustering (Bray-Curtis PCoA)**

PCoA based on Bray-Curtis dissimilarity showing all samples with technical replicates (GU54M6-R1, R2, R3; red) highlighted. The red polygon connects the three replicate samples. The mean pairwise Bray-Curtis distance among technical replicates was 0.057, compared to a mean biological distance of 0.61 across non-replicate samples a 10.7-fold difference confirming negligible pipeline-introduced variation. Dashed ellipses represent 95% confidence intervals by time point.

**Figure S16. Technical vs Biological Bray-Curtis Variation**

Boxplot comparing pairwise Bray-Curtis distances within technical replicates (n=3 pairs; red) versus biological samples (all non-replicate pairwise distances; dark blue). Technical replicate distances (mean = 0.057) are substantially lower than biological distances (mean = 0.61), confirming that variation introduced by the DNA extraction and sequencing pipeline is negligible relative to true biological microbiome differences.

**Figure S17. Differential abundance analysis between healthy controls and prediabetic baseline.**

Linear Discriminant Analysis Effect Size (LEfSe) bar plot identifying differentially abundant gut microbiota at the genus level between healthy controls (HC, ) and prediabetic participants at baseline (1-day, ). Features are sorted by their Linear Discriminant Analysis (LDA) score (), with taxa enriched in the prediabetic baseline shown in dark blue (top) and taxa enriched in healthy controls shown in green (bottom). Statistical significance was determined using a Kruskal-Wallis test () with an effect size threshold of LDA score . The clear separation highlights a distinct baseline dysbiosis in the prediabetic cohort prior to any dietary intervention.

**Supplementary Results-R**

**Integrated Network Analysis**

To investigate cross-omics interactions during Ramada fasting, we used a *MintTea*-based integrated network analysis, linking microbial genera with predicted metabolic pathways across time intervals. This approach identified three overarching consensus modules, in which existed co-occurrence patterns of taxa as well as pathways, a subset of which demonstrated strong discriminative performance (AUROC > 0.90) (Figure S11,a-b).

**1-day vs. 1-month**

Two major modules were indentified in the early fasting phase. Module 1 was characterized by associations between gallate catabolic pathways (I and II) and *Klebsiella* genus, with moderate prediction accuracy (AUROC ~0.76). In contrast, Module 2 formed a compact cluster linking lipid-related pathways including oleate and palmitoleate biosynthesis and fatty acid biosynthesis superpathway, with E. coli to *Dialister, Dorea,* and *Prevotella_9* genera. This module showed high discriminative ability (AUROC ~0.92), reflecting a rapid reorganization of microbial fatty lipid metabolism after one month of fasting.

**1-day vs. 3-month**

Longitudinal integration revealed a strong Module 1 that is enriched in nucleotide and one-carbon metabolic processes. Specifically, formaldehyde assimilation II (RuMP cycle), formaldehyde oxidation I, and pyrimidine nucleotide biosynthesis pathways showed a tight clustering together with genera such as *Blautia, Collinsella, Fusicatenibacter,* and *Eubacterium hallii group*. This module consistently achieved high predictive accuracy (AUROC ~0.94), indicating indicating coordinated functional restructuring over longer fasting durations.

**1-month vs. 3-month**

The shift from 1-month to 3-month fasting yielded three modules. Module 1 linked amino acid biosynthesis, i.e., L-lysine biosynthesis II, and carbohydrate catabolic processes, i.e., lactose and galactose breakdown I, to *Coprococcus and Holdemanella-like* lineages (AUROC ~0.79). Module 2 was dominated by fatty acid biosynthesis pathways (stearate, oleate, palmitoleate) and the polyamine biosynthesis superpathway, co-occurring with *Prevotella_9, Dialister, Sutterella, UCG-002,* and *Lachnospiraceae NK4A136 group* (AUROC ~0.90). Conversely, Module 3 clustered lineages such as *Klebsiella, Phascolarctobacterium, Streptococcus.*

Overall, these results demonstrate that Ramadan fasting generated time-specific cross-omics modules. with early intervals dominated by lipid-associated signatures (1-day vs. 1-month), intermediate-to-late intervals characterized by coordinated nucleotide and one-carbon metabolic processes (1-day vs. 3-month), and later intervals reflecting combined shifts in fatty acid biosynthesis and polyamine-associated pathways (1-month vs. 3-month).
