# Supplementary material for: Ramadan fasting induces temporal gut microbiome remodeling without metabolic improvement in adults with prediabetes
Source: Microbiol Spectr. 2026 Aug 3;14(9):e00742-26. doi: 10.1128/spectrum.00742-26 (PMC13532256; doi:10.1128/spectrum.00742-26)
Supplement: Supplementary file 2 [file spectrum.00742-26-s0003.docx]

**Supplementary File_S2_Supporting Methods**

**Ramadan Fasting Is Associated with Temporal Gut Microbiome Remodeling Without Metabolic Improvement in Adults with Prediabetes**

Ihsan Boyaci^1^, Busranur Delice^2^, Fatma Koc^2&^, Suleyman Yildirim^2,3^*

^1^Istanbul Medipol University, Vatan Clinic, Istanbul, 34214, Türkiye.

^2^Department of Medical Microbiology, Istanbul Medipol University International School of Medicine, Istanbul, Türkiye.

^3^Regenerative and Restorative Medicine Research Center, REMER, Istanbul Medipol University & Research Institute for Health Sciences and Technologies (SABITA), Istanbul Medipol University, Istanbul, Türkiye.

**^*^**Corresponding author

E-mail: [suleymanyildirim@medipol.edu.tr](mailto:suleymanyildirim@medipol.edu.tr)

^&^Current Address: APC Microbiome Ireland, University College Cork, Ireland

**MATERIALS AND METHODS**

**Collection of Samples and Biochemical Analyses**

Blood and stool samples were collected from participants at three predefined time points: within three days before Ramadan (baseline; day 1), during the last three days of Ramadan (1-month), and three months after the end of Ramadan (3-month time point). Participants were instructed to avoid major dietary changes throughout the study period and to collect stool samples using sterile collection kits provided by the research team.

Venous blood samples were collected into 8.5 mL serum separator tubes (Becton Dickinson, Franklin Lakes, NJ, USA), while samples for glycated hemoglobin (HbA1c) analysis were collected into 2 mL EDTA tubes. All specimens were stored at -80 °C until analysis. Glycated hemoglobin (HbA1c) was measured using boronate affinity chromatography on the Quo-Lab® analyzer (EKF Diagnostics PLC, Cardiff, UK) and expressed as a percentage according to the National Glycohemoglobin Standardization Program (NGSP).

Plasma glucose and fasting plasma glucose (FPG) concentrations were measured using the hexokinase method on a Roche Cobas Integra® 400 Plus analyzer with the GLUC kit (Roche Diagnostics, Mannheim, Germany). Participants meeting criteria for impaired fasting glucose and/or impaired glucose tolerance underwent a standard 2 h 75 g oral glucose tolerance test.

Total cholesterol (T-col), triglycerides (TG), high-density lipoprotein cholesterol (HDL-c), and low-density lipoprotein cholesterol (LDL-c) were measured on a Roche Cobas Integra® 400 Plus analyzer using the CHOL2, TRIGL, HDLC4, and LDL-C3 kits, respectively, employing enzymatic colorimetric methods (Roche Diagnostics, Mannheim, Germany). Urinary albumin was measured on the same analyzer using the ALBT2 kit with an immunoturbidimetric method (Roche Diagnostics, Mannheim, Germany). Estimated glomerular filtration rate (GFR) was calculated using the Chronic Kidney Disease Epidemiology Collaboration (CKD-EPI) equation. Fasting insulin and C-peptide concentrations were measured on a Roche Cobas e601 analyzer using the Elecsys Insulin and Elecsys C-peptide assays with electrochemiluminescence immunoassay (ECLIA) methodology. C-peptide was assessed to provide additional validation of pancreatic β-cell function and endogenous insulin secretion capacity, as it is not subject to hepatic first-pass metabolism and offers a more stable indicator of insulin production compared with insulin alone. The combined assessment of insulin resistance using the homeostatic model assessment of insulin resistance (HOMA-IR) and C-peptide enabled a more comprehensive evaluation of insulin resistance and β-cell function during the progression from normal glucose tolerance to prediabetes. Insulin resistance was calculated using the homeostatic model assessment of insulin resistance (HOMA-IR) index: HOMA-IR = (fasting insulin (μIU/mL) × fasting glucose (mmol/L)) / 22.5 (1).

**Recovery of Stool DNA and Sequencing 16S rRNA Genes**

Fecal samples were obtained in sterile stool holders with built-in collection spatulas to reduce contamination. Participants received a sterile collection paper to maintain sample integrity during defecation. Samples were stored directly at −80 °C until extraction of genomic DNA. Genomic DNA was extracted using the QIAamp Fast DNA Stool Mini Kit (Qiagen, Hilden, Germany), in accordance with the manufacturer's protocol, with an added mechanical lysis step using 0.1 mm zirconium beads (Sigma-Aldrich, St. Louis, MO, USA), and bead beating performed using a Next Advance Bullet Blender to improve recovery of microbial DNA. The procedure involved a lysozyme incubation step (20 mg/mL, 37 °C, 10 min), and then proteinase K, RNase A, and AL buffer were added, after which purification was carried out by ethanol precipitation and by column purification. The DNA concentrations were quantified by means of the Qubit dsDNA HS Assay Kit (Thermo Fisher Scientific, USA) in a Qubit 2.0 fluorometer. DNA integrity was examined by agarose gel electrophoresis, and only samples meeting quality thresholds were processed further.

Hypervariable regions V3-V4 of 16S rRNA genes were amplified by primers 341F (CCTACGGGNGGCWGCAG) and 805R (GACTACHVGGGTATCTAATCC). The 25 μL PCR reactions consisted of 12.5 μL 2× KAPA HiFi HotStart ReadyMix (Roche Sequencing Solutions, Wilmington, MA, USA), 0.5 μL each of primers 10 μM, and 20 ng of template DNA in each reaction. The thermal cycle process was initiated at 95 °C for 3 minutes during denaturation, then 20 times 30 seconds at 95 °C, 30 seconds at 55 °C, and 30 seconds at 72 °C, and then terminated by 72 °C extension for 5 minutes. The amplicons were then verified by agar gel electrophoresis, purified by Agencourt AMPure XP system (Beckman Coulter, USA), and quantified. Negative extraction controls and no-template PCR controls were collected for each run and included in the sequencing library.

Libraries were normalized to 6 nM, pooled, and denatured with 0.2 N NaOH. Sequencing was performed on the Illumina MiSeq platform using the MiSeq Reagent Kit v2 (500 cycles), with 20% PhiX spike-in to improve sequence diversity and monitor run performance. Raw sequencing data were deposited in the NCBI Sequence Read Archive (accession number: PRJNA1329139).

**Microbial Community Structure and Diversity Analyses**

Alpha diversity was assessed at the genus level using Observed richness, Chao1, Shannon, and Simpson indices. To account for the longitudinal study design with repeated measurements from the same individuals, differences in alpha diversity across fasting intervals (1-day, 1-month, and 3-month) were evaluated using Friedman repeated measures tests. When the overall test was significant, post hoc pairwise comparisons were performed using paired Wilcoxon signed-rank tests with false discovery rate (FDR) correction for multiple testing. Alpha diversity indices were calculated using unfiltered count data to avoid bias in richness estimators that depend on rare taxa.

Beta diversity was computed using the Bray-Curtis dissimilarity matrix. Community-level differences were visualized using Principal Coordinates Analysis (PCoA) and Canonical Analysis of Principal Coordinates (CAP). Differences in overall community composition across fasting intervals were tested using PERMANOVA (adonis2 function in the vegan package, 999 permutations), with permutations constrained within individuals (strata = subject ID) to account for repeated measurements. Pairwise PERMANOVA comparisons between fasting intervals were performed using subject-blocked permutations and FDR correction. Homogeneity of multivariate dispersion was assessed using the betadisper function followed by permutation testing to verify PERMANOVA assumptions. Hierarchical clustering was conducted on Bray-Curtis distance matrices using the hclust function, and the statistical significance of cluster separation was evaluated using permutation-based approaches. Temporal trajectories of selected microbial taxa were examined using locally estimated scatterplot smoothing (LOESS) regression and visualized using the ggplot2 package.

To contextualize the prediabetic microbiome, seventeen healthy adult controls (HC; n=17; matched for age and sex) were incorporated as a baseline reference group from our previously published cohort (2). HC samples were processed in a separate sequencing run from the prediabetic cohort. To address potential between-batch technical variation, ConQuR (conditional quantile regression; v1.0) (3) was applied for batch effect correction, with the prediabetic batch designated as the reference. PERMANOVA confirmed that the Batch variable explained 0% of compositional variance (R² = 0, Df = 0), indicating that the biological Group effect (R² = 0.174, p = 0.001) was the dominant source of variance and that no measurable technical batch confounding was present. Beta diversity differences between HC and prediabetic baseline (1-day) were assessed using PERMANOVA (999 permutations, Type III) with age and sex as covariates. Alpha diversity comparisons between HC and all prediabetic time points were performed using Wilcoxon rank-sum tests with BH correction, and overall multi-group comparisons used Kruskal-Wallis tests with Dunn post-hoc testing.

Indicator taxa were identified using both linear discriminant analysis effect size (LefSe) (LDA > 2.5) and indicator species analysis. Associations between microbial taxa and host metabolic parameters were assessed using biweight midcorrelation (bicor; WGCNA). Analyses were performed separately for each fasting interval (1-day, 1-month, and 3-month), using all available observations. Multiple testing was controlled within each time interval using false discovery rate (FDR) correction, and associations with |r| ≥ 0.35 and FDR-adjusted p < 0.10 were considered significant. Given the longitudinal nature of the study, analyses were conducted using all available observations, and statistical approaches robust to unbalanced repeated measures were applied where appropriate. Additionally, core microbiome analyses were carried out using the microbiome package to identify taxa with prevalence >50% across fasting intervals.

All analyses and visualizations were conducted in R (v.4.2.3) using packages including phyloseq, vegan, indicspecies, ggplot2, pheatmap, lattice, microbiome, WGCNA, and custom scripts.

**Multivariable and Network-Based Analyses**

To identify microbial features associated with clinical and temporal variables, we employed MaAsLin2 (4) (Microbiome Multivariable Association with Linear Models, v1.10.0) in R. Genus-level relative abundance data were used as input, and features were filtered to exclude those present in fewer than 10% of samples. Continuous metadata variables included age, BMI, HbA1c, HOMA-IR, fasting insulin, and lipid parameters (HDL, LDL, triglycerides, total cholesterol). Categorical metadata included time intervals (day 1, 1 month, and 3 months).

MaAsLin2 was run with a default linear model framework, using arcsine square-root transformation for relative abundance data. Significance was assessed with Benjamini-Hochberg FDR correction, and features with q < 0.25 were considered significant. Model diagnostics and residual checks were performed to ensure assumptions were met.

Cross-omics interactions between microbial taxa and predicted functional pathways were explored using the MintTea framework (5). Preprocessing involved total sum scaling (TSS) normalization and filtering of rare features (<15% non-zero abundance). We applied 100 iterations of 80% subsampling across the datasets. In each iteration, sparse Generalized Canonical Correlation Analysis (sGCCA) was used to identify consensus co-occurrence modules.

Modules were evaluated by calculating the first principal component (PC1) of each module and estimating its discriminative power using the area under the ROC curve (AUROC). Only modules with AUROC > 0.70 and greater than random expectations were retained. Cross-omics correlations within modules were quantified using the biweight midcorrelation (bicor function, WGCNA R package).

For visualization, consensus networks were exported and rendered in Gephi (v0.10.1), where nodes represented microbial genera or pathways and edges indicated robust co-occurrence (>80% of subsampling iterations). Module identity and connectivity patterns were annotated for each fasting interval comparison (day 1 vs. 1 month, day 1 vs. 3 months, and 1 month vs. 3 months).

**REFERENCES**

1.	Matthews DR, Hosker JP, Rudenski AS, Naylor BA, Treacher DF, Turner RC. 1985. Homeostasis model assessment: insulin resistance and beta-cell function from fasting plasma glucose and insulin concentrations in man. Diabetologia 28:412–419.

2.	Arikan M, Yildiz Z, Kahraman Demir T, Yilmaz NH, Sen A, Hanoglu L, Yildirim S. 2022. Axillary Microbiota Is Associated with Cognitive Impairment in Parkinson’s Disease Patients. Microbiol Spectr 10.

3.	Ling W, Lu J, Zhao N, Lulla A, Plantinga AM, Fu W, Zhang A, Liu H, Song H, Li Z, Chen J, Randolph TW, Koay WLA, White JR, Launer LJ, Fodor AA, Meyer KA, Wu MC. 2022. Batch effects removal for microbiome data via conditional quantile regression. Nature Communications 2022 13:1 13:5418-.

4.	Mallick H, Rahnavard A, McIver LJ, Ma S, Zhang Y, Nguyen LH, Tickle TL, Weingart G, Ren B, Schwager EH, Chatterjee S, Thompson KN, Wilkinson JE, Subramanian A, Lu Y, Waldron L, Paulson JN, Franzosa EA, Bravo HC, Huttenhower C. 2021. Multivariable association discovery in population-scale meta-omics studies. PLoS Comput Biol 17:e1009442.

5.	Muller E, Shiryan I, Borenstein E. 2024. Multi-omic integration of microbiome data for identifying disease-associated modules. Nat Commun 15:2621.
